# Features of tumor-microenvironment images predict targeted therapy survival benefit in patients with *EGFR*-mutant lung cancer

**DOI:** 10.1172/JCI160330

**Published:** 2023-01-17

**Authors:** Shidan Wang, Ruichen Rong, Donghan M. Yang, Junya Fujimoto, Justin A. Bishop, Shirley Yan, Ling Cai, Carmen Behrens, Lynne D. Berry, Clare Wilhelm, Dara Aisner, Lynette Sholl, Bruce E. Johnson, David J. Kwiatkowski, Ignacio I. Wistuba, Paul A. Bunn, John Minna, Guanghua Xiao, Mark G. Kris, Yang Xie

**Affiliations:** 1 Quantitative Biomedical Research Center, The Peter O’Donnell Jr. School of Public Health, University of Texas Southwestern Medical Center, Dallas, Texas, USA.; 2 Department of Translational Molecular Pathology, Division of Pathology/Lab Medicine, University of Texas MD Anderson Cancer Center, Houston, Texas, USA.; 3 Department of Pathology, University of Texas Southwestern Medical Center, Dallas, Texas, USA.; 4 Department of Biostatistics, Vanderbilt University Medical Center, Nashville, Tennessee, USA.; 5 Department of Thoracic Oncology, Memorial Sloan Kettering Cancer Center, New York, New York, USA.; 6 Department of Pathology, University of Colorado, Denver, Colorado, USA.; 7 Department of Pathology, Brigham and Women’s Hospital, Harvard University, Boston, Massachusetts, USA.; 8 Department of Medicine, Brigham and Women’s Hospital, Boston, Massachusetts, USA.; 9 Department of Medical Oncology, Dana-Farber Cancer Institute, Harvard University, Boston, Massachusetts, USA.; 10 Division of Medical Oncology, School of Medicine, University of Colorado, Aurora, Colorado, USA.; 11 Hamon Center for Therapeutic Oncology Research,; 12 Departments of Internal Medicine and Pharmacology,; 13 Simmons Comprehensive Cancer Center, and; 14 Department of Bioinformatics, UT Southwestern Medical Center, Dallas, Texas, USA.

**Keywords:** Oncology, Lung cancer

## Abstract

Tyrosine kinase inhibitors (TKIs) targeting epidermal growth factor receptor (EGFR) are effective for many patients with lung cancer with *EGFR* mutations. However, not all patients are responsive to EGFR TKIs, including even those harboring *EGFR*-sensitizing mutations. In this study, we quantified the cells and cellular interaction features of the tumor microenvironment (TME) using routine H&E-stained biopsy sections. These TME features were used to develop a prediction model for survival benefit from EGFR TKI therapy in patients with lung adenocarcinoma and *EGFR*-sensitizing mutations in the Lung Cancer Mutation Consortium 1 (LCMC1) and validated in an independent LCMC2 cohort. In the validation data set, EGFR TKI treatment prolonged survival in the predicted-to-benefit group but not in the predicted-not-to-benefit group. Among patients treated with EGFR TKIs, the predicted-to-benefit group had prolonged survival outcomes compared with the predicted not-to-benefit group. The EGFR TKI survival benefit positively correlated with tumor-tumor interaction image features and negatively correlated with tumor-stroma interaction. Moreover, the tumor-stroma interaction was associated with higher activation of the hepatocyte growth factor/MET-mediated PI3K/AKT signaling pathway and epithelial-mesenchymal transition process, supporting the hypothesis of fibroblast-involved resistance to EGFR TKI treatment.

## Graphical abstract

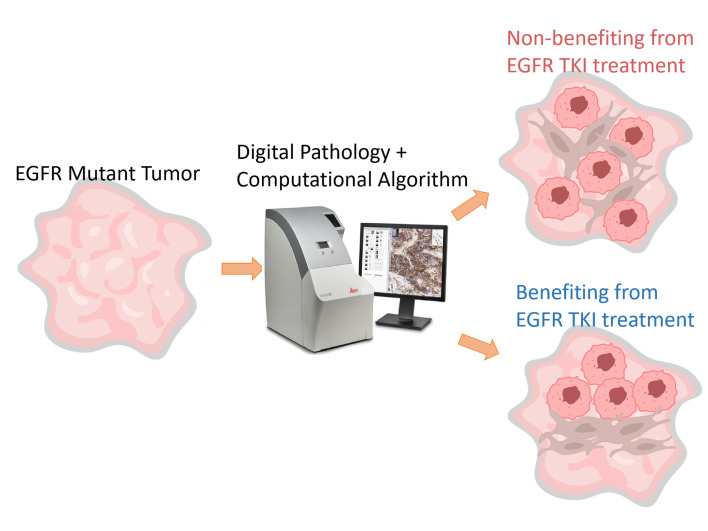

## Introduction

Tyrosine kinase inhibitors (TKIs) of the epidermal growth factor receptor (EGFR) have shown survival improvements in the treatment of patients with *EGFR-*mutant lung cancer as first line therapy. Erlotinib, an EGFR TKI, was also the first globally approved targeted therapy for locally advanced or metastatic non–small cell lung cancer (NSCLC) (1, 2). Response rates for EGFR TKI therapy range from 60%–80% in patients with NSCLC carrying sensitizing *EGFR* mutations (3–5). This leaves a substantial portion of unresponsive cancers, making it clinically important to prospectively identify predictors of response and resistance to EGFR TKIs.

As a routine clinical procedure, H&E-stained pathology tissue slides provide detailed tumor morphological characterization at high resolution. Multiple studies have explored the relationship between clinically defined pathological subtypes and targeted therapy response. Kim et al. reported the dominant papillary subtype as predictive for EGFR TKI sensitivity in patients with lung adenocarcinoma (LUAD) (6); Miller et al. reported the bronchioloalveolar pathologic subtype — which may represent several different growth patterns today — as associated with EGFR TKI efficacy in patients with NSCLC (7); and Yatabe et al. observed the overlapping characteristics between the bronchioloalveolar subtype and terminal-respiratory-unit type LUAD, for which the *EGFR* mutation is specific (8). However, there is a lack of objective quantification, independent validation, and biological characterization of histopathological features and their predictive value in the context of response to EGFR TKI. With the development of whole-slide image scanning techniques and deep-learning based image analysis methods, (9) computational analysis of pathology images has tremendous potential to assist pathologists with cancer diagnosis and prognosis (10). Therefore, there is a need to investigate the potential use of sophisticated pathology image analysis approaches to predict response to EGFR TKIs.

In this study, we first applied a published deep-learning based analysis algorithm, histology-based digital-staining (HD-staining) method (11), to classify cell types in standard H&E-stained pathology images. Then, we characterized and quantified the tumor microenvironment (TME) based on cell-type densities and cellular interactions, which are attributes associated with survival and genomic features of patients with LUAD (11). Next, the extracted image features were used to develop and validate a prediction model for survival benefit from EGFR TKIs in patients with *EGFR*-mutant LUAD using 2 independent clinical trial cohorts. Gene expression analysis was used in a third independent cohort to examine potential molecular mechanisms of resistance suggested by the risk prediction model.

## Results

### TME image features predict EGFR TKI survival benefit.

**Figure 1:**
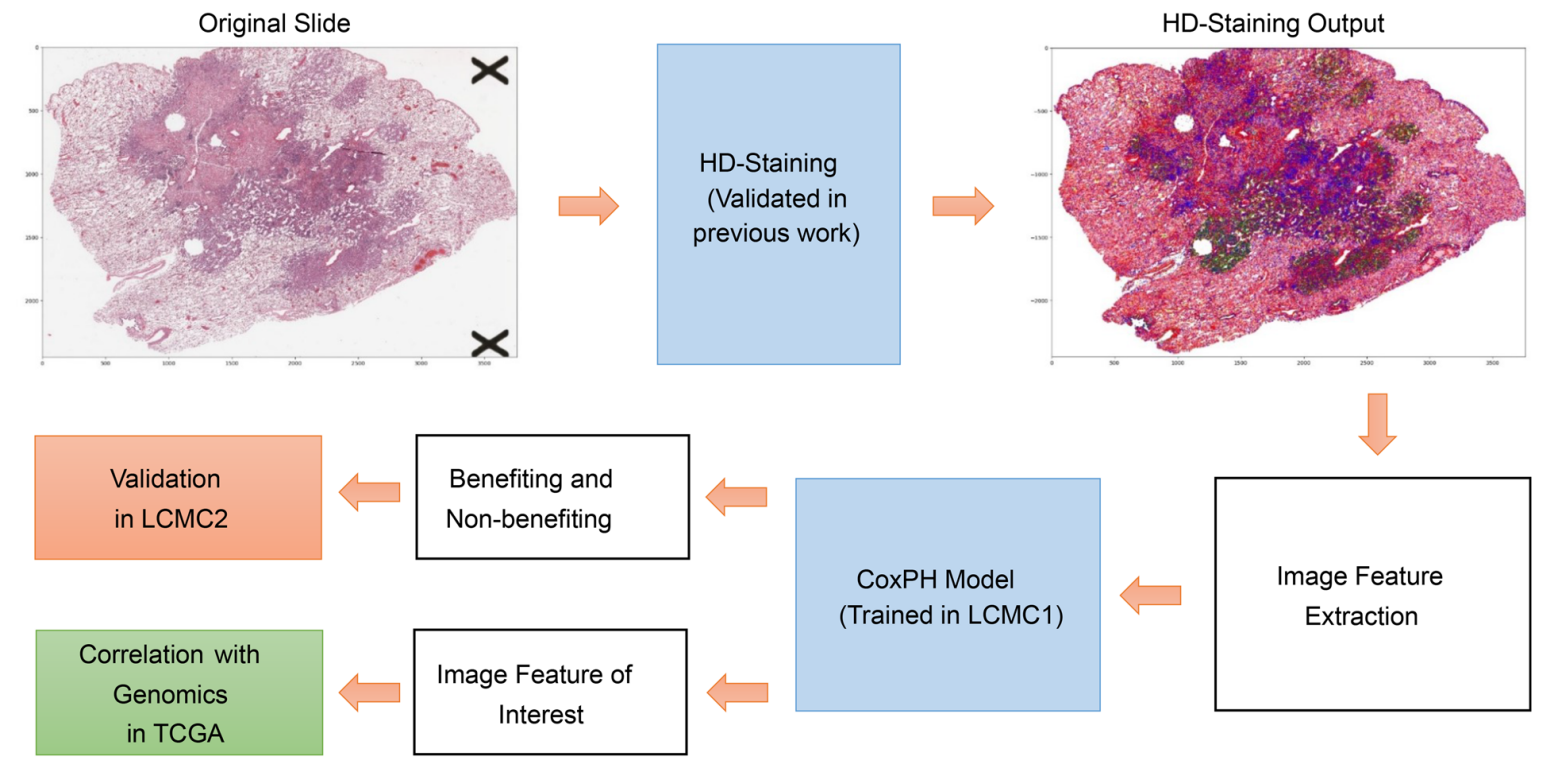
Flowchart of developing and validating computational staining-based model to predict EGFR TKI survival benefit. HD-staining is a previously described image analysis pipeline (11). CoxPH, Cox proportional hazards; LCMC, Lung Cancer Mutation Consortium; TCGA, The Cancer Genome Atlas.

A training set of pretreatment H&E-stained pathology images was derived from 168 patients with sensitizing *EGFR* mutations enrolled in the multi-institutional Lung Cancer Mutation Consortium 1 (LCMC1) (12). Corresponding clinical data for 150 patients with *EGFR*-mutant metastatic LUAD who received EGFR TKI treatment were also collected. The TME was characterized using 12 image features representing densities of different cell types and their established interactions with tumor cells via a previously described pathology image analysis pipeline (11) (Figure 1). Image features included tumor nuclei density, stroma nuclei density, lymphocyte density, red blood cell density, macrophage density, karyorrhexis density, tumor-tumor interaction, tumor-stroma interaction, tumor-lymphocyte interaction, tumor–red blood cell interaction, tumor-macrophage interaction, and tumor-karyorrhexis interaction, where the interaction between tumor and a specific cell type was defined as the proportion of the cell type in all cells surrounding tumor cells (Supplemental Table 1; supplemental material available online with this article; https://doi.org/10.1172/JCI160330DS1). A penalized Cox proportional hazards prediction model (13) was used to select image features associated with overall survival (OS) of patients with *EGFR*-mutant LUAD who received EGFR TKI treatment from the LCMC1 training set. Of the 12 image features, 2 showed tumor-tumor interaction and tumor-stroma interaction correlated with survival benefit from TKI therapy in the LCMC1 training set and were selected by the model (Supplemental Table 1 and Supplemental Figure 1). Tumor-tumor interaction correlated with prolonged OS following EGFR TKI therapy (lower risk score; per 10%, HR = 0.73, 95% CI 0.58–0.90, *P* = 0.004), while tumor-stroma interaction negatively correlated with OS (higher risk score; per 10%, HR = 1.53, 95% CI 1.11–2.10, *P* = 0.009).

### Validation of the EGFR TKI survival benefit prediction model.

**Figure 2:**
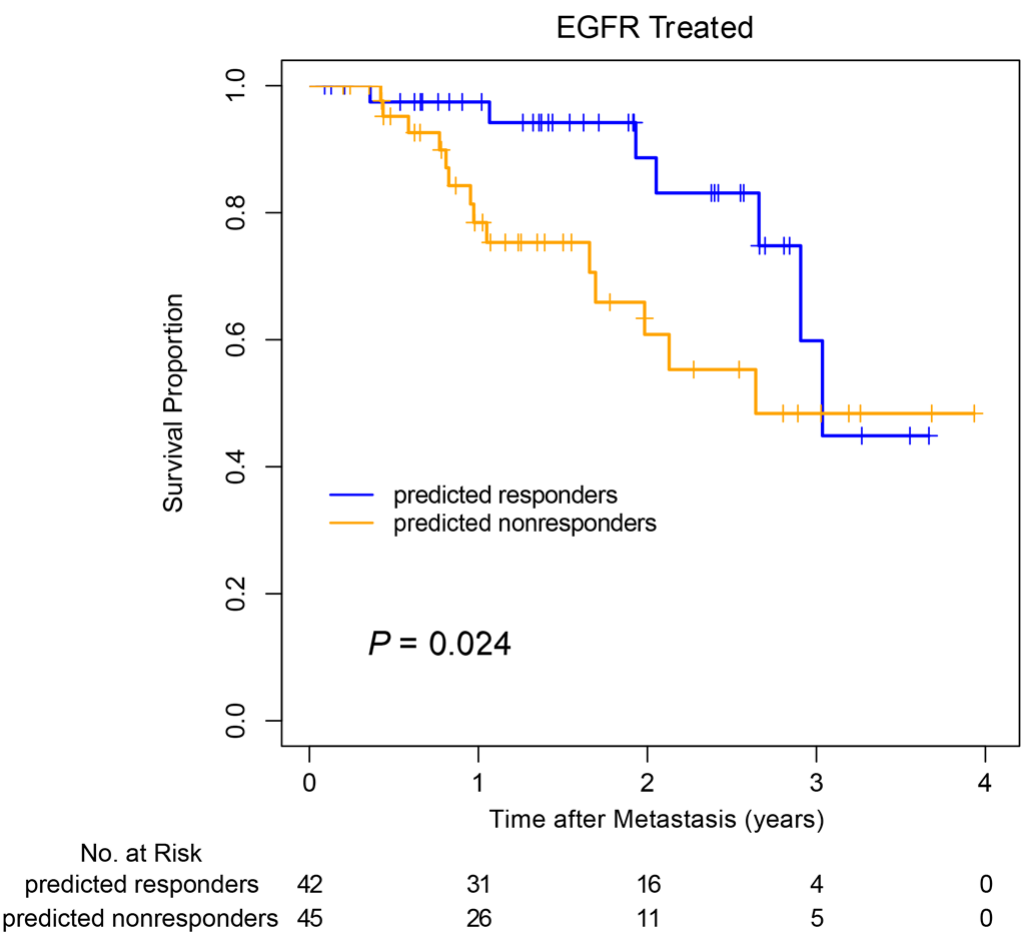
Validation of image feature-based EGFR TKI survival benefit prediction model. All patients carried sensitizing *EGFR* mutations. Survival curves of predicted-to-benefit and predicted-not-to-benefit groups in the EGFR TKI treated LCMC2 validation data set were plotted using the Kaplan-Meier method with the log-rank test.

The prediction model was then validated using an independent data set from the LCMC2 cohort including 122 patients with *EGFR-*mutant metastatic LUAD, comprising both sensitizing and other *EGFR* mutation types; 88 were treated with EGFR TKI, and 34 did not receive an EGFR TKI treatment. The same penalized Cox prediction model developed from the LCMC1 training cohort was applied to the LCMC2 cohort to calculate risk scores for each patient — a higher risk score indicated nonbenefit from TKI treatment. Then, patients in the LCMC2 cohort were divided into 2 groups using a median split of risk scores, EGFR TKI predicted-to-benefit or predicted-not-to-benefit groups. Among the 87 patients who received EGFR TKI therapy and had available OS, the 42 patients in the predicted-to-benefit group showed significantly better OS than the 45 patients in the predicted-not-to-benefit group (Figure 2, *P* = 0.024).

**Figure 3:**
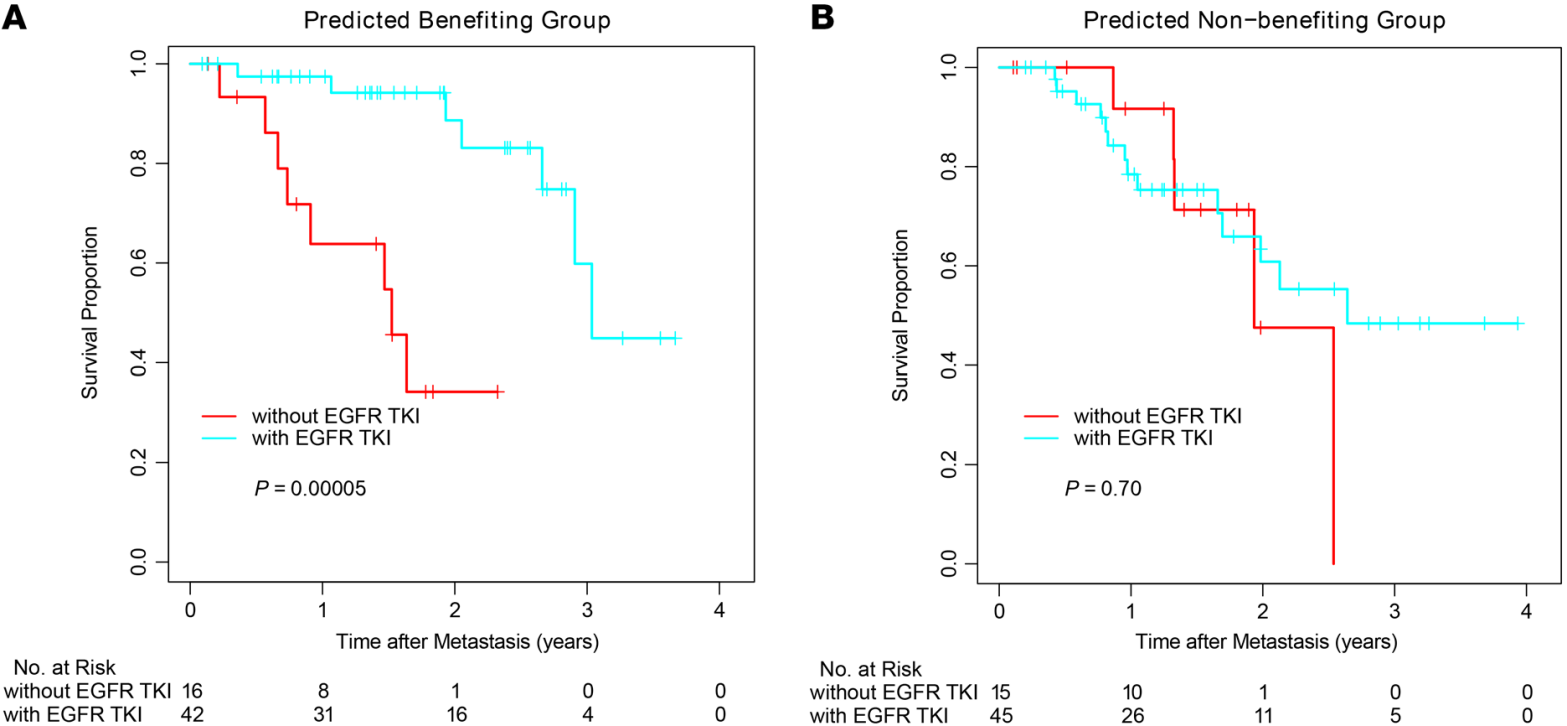
Predictive value of image feature-based EGFR TKI survival benefit prediction model. Kaplan-Meier (KM) plots of patients with tumors harboring *EGFR* mutations in the predicted-to-benefit group (**A**) or predicted-not-to-benefit group (**B**) to EGFR TKI treatment in the LCMC2 data set. *P* values were estimated with the log-rank test.

To further investigate predictive performance, the Cox prediction model was applied to 118 patients with both *EGFR* mutations and available OS in the LCMC2 cohort. Among the predicted-to-benefit group, 16 patients who did not receive EGFR TKI therapy showed significantly worse OS than the 42 patients who received EGFR TKI therapy (Figure 3A, *P* < 0.001; HR = 9.05, 95% CI 2.57–31.93). In contrast, EGFR TKI treatment did not affect OS within the predicted-not-to-benefit group (Figure 3B, n = 60 [45 received EGFR TKI therapy and 15 did not], *P* = 0.70; HR = 1.26, 95% CI 0.44–3.57). Furthermore, after adjusting for potential clinical confounders, including age, sex, smoking status, and surgery, the interaction between EGFR TKI therapy and predicted group was still significant (Table 1, *P* = 0.046). The predictive value of the risk prediction models was still observed in patients with *EGFR*-sensitizing mutations by further stratifying patient groups according to EGFR mutation type (Supplemental Figure 2; did-not-receive versus received EGFR TKI therapy among the predicted-to-benefit group with sensitizing-EGFR *(sEGFR*) mutations, *P* < 0.001, HR = 18.33, 95% CI 3.30–101.73; among predicted-not-to-benefit group with *sEGFR* mutations, *P* = 0.80, HR = 1.19, 95% CI 0.26–5.40).

**Figure 4:**
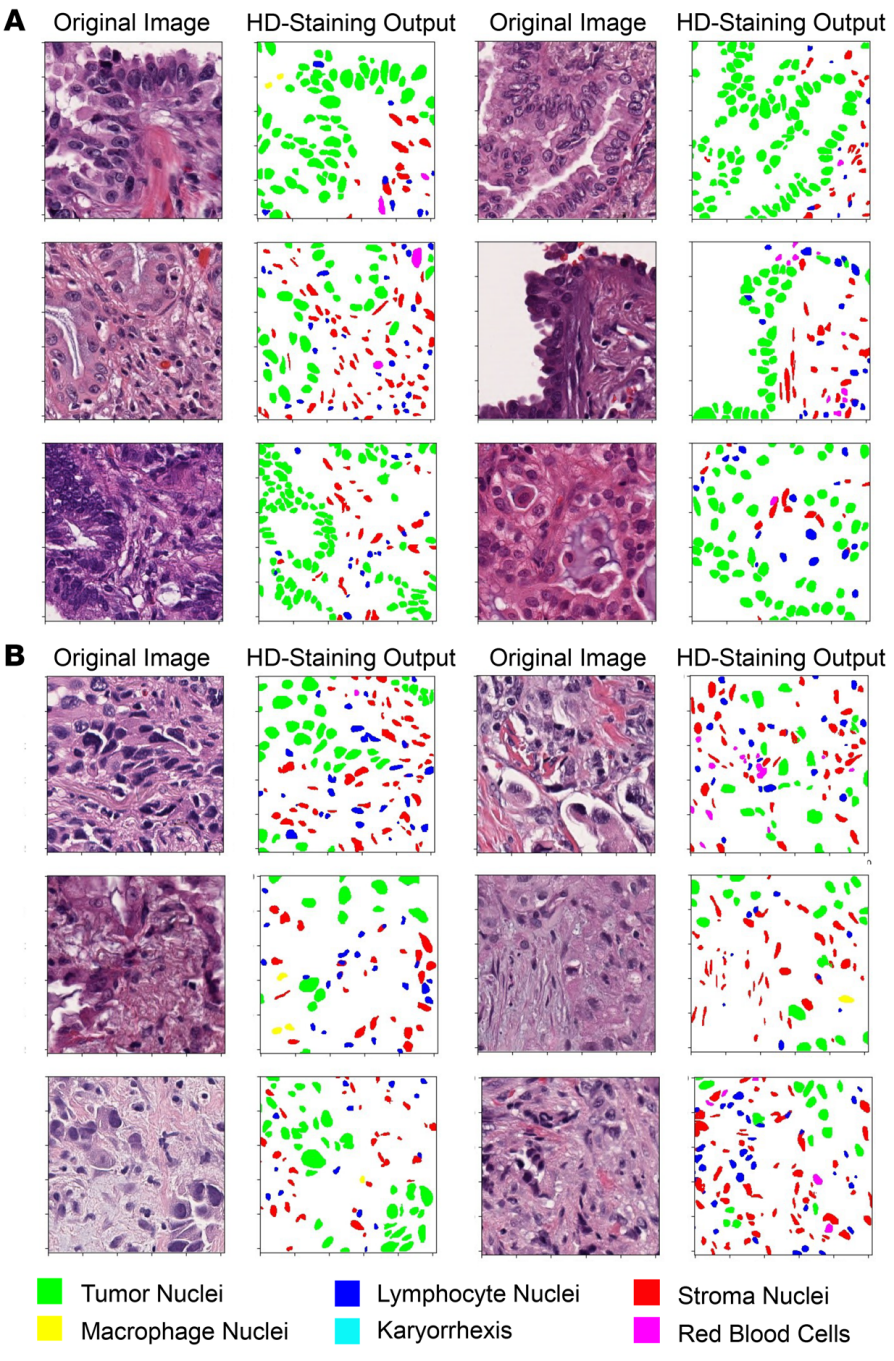
Representative pathology images and analysis pipeline output. H&E-stained images (columns 1 and 3) and HD-staining analysis output (columns 2 and 4) of predicted EGFR TKI predicted-to-benefit group (**A**) and predicted-not-to-benefit group (**B**) from the LCMC2 data set.

Consistent with the coefficients from the prediction model (Supplemental Table 1 and Supplemental Figure 1), pathological images show that there were more proliferative tumor cells (tumor-tumor interactions) from patients in the predicted-to-benefit group (Figure 4A), while there were more tumor-stroma interactions in the predicted-not-to-benefit group (Figure 4B). Moreover, the association between predicted group and LUAD subtypes annotated by pathologists shows significant correlation between lack of survival benefits from EGFR TKI therapy and solid subtype (Supplemental Table 2, *P* < 0.001 in both the training set from the LCMC1 cohort and validation set from the LCMC2 cohort). The observation is also consistent with a recent report of correlation between high-grade patterns and EGFR TKI resistance in patients with relapsed lung cancer (14). The image features extracted from digital pathology image analysis have captured more information than LUAD subtypes annotated by pathologists. To connect the predictive image features with additional prior pathology knowledge, closer inspection of images together with predictive features and outcomes (Supplemental Table 3) found that predictive image features are related to the tumor/stroma ratios (TSR), which has been shown to correlate with poor outcomes in solid tumors (15). We then calculated TSR by calculating the number of tumor cells divided by the number of stroma cells within the tumor region of interest for each individual slide. Additional analysis shows that TSR correlated with the risk scores and the predicted groups for EGFR TKI therapy calculated by predictive image features (*P* < 0.001, Supplemental Figure 3, A and B). However, TSR is not significantly associated with survival outcome in the multivariate analysis (Supplemental Figure 3C).

### Pathological image features correlate with biological pathway activation.

**Figure 5:**
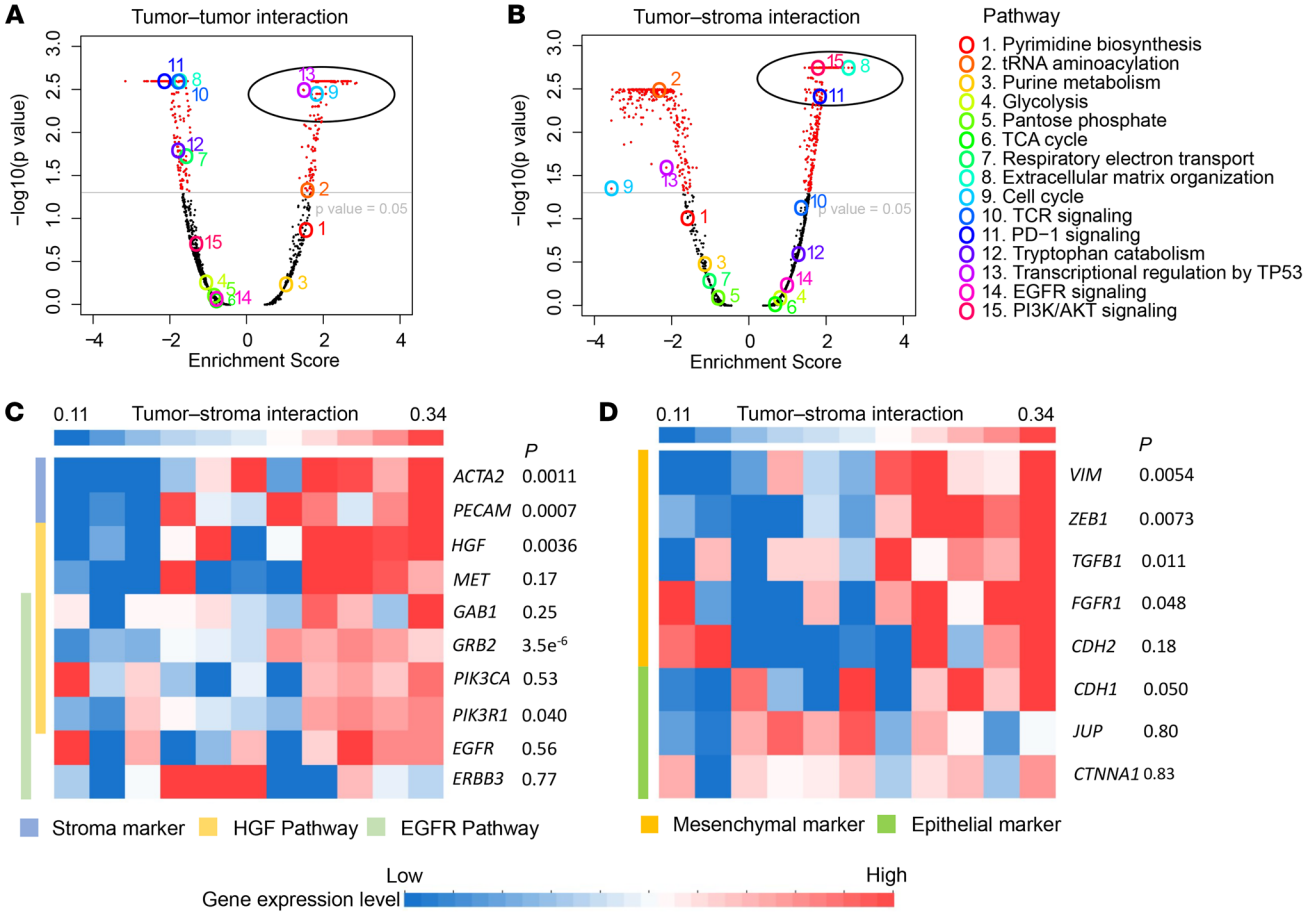
Correlation between EGFR TKI survival benefit-predictive image features and mRNA expression in tumors with *EGFR* mutation. (**A** and **B**) Volcano plots of gene set enrichment analysis results correlating mRNA expression level with tumor-tumor interaction (**A**) and tumor-stroma interaction (**B**). Colored open circles represent 15 reactome gene sets of interest. Filled circles depict mRNA expression levels. Black, nonsignificant; red, significant. (**C** and **D**) Gene-expression heatmap depicting relationships between tumor-stroma interactions and mRNA expression of genes of interest based on GSEA analysis. Patients with *EGFR* mutations in the TCGA data set were grouped and sorted according to tumor-stroma interaction, as each column depicts 1 patient group. (**C**) Markers for fibroblast cells (light blue in side bar), genes involved in HGF-induced PIP3 activation (light yellow), and genes involved in EGFR-induced PIP3 activation (light green) were selected. (**D**) Classic EMT marker genes were selected. Side bar: yellow, mesenchymal markers; green, epithelial markers. HGF, hepatocyte growth factor.

To investigate whether molecular mechanisms underlie the predictive value of image-based features for EGFR TKI therapy survival benefit, association analysis between image features and mRNA expression was performed for patients with LUAD (*n* = 53) harboring EGFR mutations in The Cancer Genome Atlas (TCGA) data set. Baseline pathological images and genome-wide transcriptome data were available for these 53 patients. Analyses were focused on identifying which biological pathway activations were associated with image features that were shown to correlate with survival benefit from EGFR TKI therapy, specifically, tumor-tumor and tumor-stroma interactions. Gene set enrichment analysis (GSEA) in the Reactome database identified multiple biological pathways whose mRNA expression profiles correlated with tumor-tumor interaction (Figure 5A; similar findings for KEGG and GO analysis in Supplemental Figure 4). The results showed that transcriptional activation of the cell cycle pathway positively correlated with increased tumor-tumor interactions and, by extension, improved survival benefit from EGFR TKI treatment. This correlation confirmed that the increased tumor-tumor interaction, as defined by image features, reflected increased tumor-cell proliferation based on molecular analysis. Interestingly, the activation of transcription regulation pathway by TP53 was also positively associated with tumor-tumor interaction. Therefore, the tumor-tumor interaction could indicate activation of TP53, a tumor suppressor whose mutation or inactivation is linked to decreased responsiveness to EGFR TKI (16). Our results of increased tumor-tumor interaction in the predicted-to-benefit group also supported the potential mechanism that proper TP53 function is required for EGFR TKI responsiveness. In summary, the GSEA results suggested image-derived tumor-tumor interaction as a biomarker for EGFR TKI responsiveness.

In contrast, transcriptional activation of extracellular organization, the PD-1 signaling pathway, and the PI3K/AKT signaling in cancer pathway positively correlated with increased tumor-stroma interactions and, by extension, EGFR TKI resistance (Figure 5B and Supplemental Figure 4). The enrichment in the extracellular organization pathway, attributed to stromal cells (17), confirms stromal infiltration into the TME for patients resistant to EGFR TKI treatment. Previous studies showed that activation of PD-1 signaling, indicative of immunosuppression, and the PI3K/AKT pathway correlates with EGFR TKI resistance (18), consistent with our observation of increased tumor-stroma interaction in the predicted-not-to benefit group.

Interestingly, EGFR signaling in cancer pathways was not enriched with either tumor-tumor interaction or tumor-stroma interaction, consistent with previous observations showing that EGFR expression is not predictive of EGFR TKI response (19). As a negative control, we randomly shuffled the patient IDs and repeated the same EGFR analysis, and the enrichment of the aforementioned pathways with either tumor-tumor interaction or tumor-stroma interaction was no longer observed (Supplemental Figure 5).

### Tumor-stroma interaction correlated with hepatocyte growth factor-phosphatidylinositol-3, 4, 5-triphosphate activation.

We next examined the individual genes contributing to the relationship between increased tumor-stroma interaction and EGFR TKI resistance. First, we hypothesized that image-derived tumor-stroma interaction could reflect molecular crosstalk between tumor cells and fibroblasts. To explore this hypothesis, we investigated the relationship between tumor-stroma interaction as quantified by our model and the expression of the stromal markers *ACTA2* — using α-smooth muscle actin (αSMA), a marker for activated fibroblast — and *PECAM1* — using CD31, a marker for angiogenesis (20). Consistent with this hypothesis, increased tumor-stroma interactions were associated with increased mRNA expression of both *ACTA2* and *PECAM1* (Figure 5C).

Next, we looked for genes upstream of the PI3K/AKT pathway, which was associated with tumor-stroma interactions (Figure 5B) and could be activated through phosphatidylinositol (3, 4, 5)-triphosphate (PIP3) activation by hepatocyte growth factor–like (HGF-like) and epidermal growth factor–like (EGF-like) ligands in parallel. Specifically, we compared expression of genes involved in HGF-mediated PIP3 activation (*HGF* and its receptor, *MET***)** with EGFR-mediated PIP3 activation (*EGFR* and *ERBB3* (21), Supplemental Figure 6); we also included *GAB1*, *GRB2*, *PIK3R1*, and *PIK3CA* that are involved in both pathways. Consistent with our observation that tumor-stroma interactions reflected activation of fibroblast cells and prior reports demonstrating that HGF secretion is predominantly attributed to stromal cells (22), *HGF* transcription was significantly correlated with tumor-stroma interaction (Figure 5C). In contrast, tumor-stroma interactions were not correlated with expression levels of *EGFR* or *EGBB3*, which are solely engaged in EGFR-mediated PIP3 activation (Figure 5C). DNA methylation analysis also showed significant negative correlation between methylation of transcriptional start site and tumor-stroma interaction, whereas the genes within the EGFR pathway have a trend of positive correlation (Supplemental Figure 7). These data suggest that activation of the PI3K/AKT pathway in EGFR TKI resistant tumors is more likely caused by HGF secretion from fibroblasts as the level of tumor-stroma interaction increased. Because EGFR TKI treatment could only suppress EGFR-mediated PI3K/AKT activation rather than HGF-mediated PI3K/AKT activation, increased HGF secretion may enable tumors to escape the inhibitory effect of EGFR TKIs and lead to drug resistance. These findings were consistent with previous cell line studies indicating that coculturing with HGF-secreting fibroblasts made *EGFR*-mutant lung cancer cell lines resistant to EGFR TKIs (23), and that ERBB3 activated the PI3K/AKT pathway in EGFR TKI sensitive — rather than resistant — NSCLC cell lines (21).

### Tumor-stroma interaction and epithelial-mesenchymal transition.

Fibroblasts are also associated with epithelial-mesenchymal transition (EMT) (24), a predictor and mechanism of EGFR TKI resistance (25–28). Therefore, we further investigated the relationship between tumor-stroma interaction and expression of genes involved in EMT. Tumor-stroma interaction correlated with expression of classic mesenchymal markers, vimentin (*VIM*), *TGFB1*, *FGFR1* (27), and *ZEB1*, a driver gene of the EMT process (29) (Figure 5D). As a comparison, we observed no significant correlation between tumor-stroma interaction and classic epithelial markers E-cadherin (*CDH1*), α-cadherin (*CTNNA1*), and γ-catenin (*JUP*, Figure 5D) (27). The correlation with transcription of mesenchymal markers may indicate activation of the EMT process for tumors with enriched tumor-stroma interactions. However, since increased tumor-stroma interactions were indicative of an accumulation of tumoral fibroblasts, the correlation may also indicate a higher proportion of fibroblasts instead of EMT. Single-cell sequencing and IHC staining could address this knowledge gap.

## Discussion

In this study, we developed a tissue image–based model to predict the survival benefit of EGFR TKI therapy in patients with *EGFR*-mutant metastatic LUAD. A previously published deep-learning algorithm, HD-staining (30), was used to quantify cell composition and cellular interactions within the TME. By analyzing patient clinical outcomes with image features, a higher tumor-tumor interaction was found to correlate with higher benefit from EGFR TKI, while a higher tumor-stroma interaction correlated with less benefit from EGFR TKI. The predictive value of this image-based model was validated in an independent cohort, in which the predicted-to-benefit group showed significantly improved OS after EGFR TKI treatment, while the predicted-not-to-benefit group did not. Although several studies have reported pathological subtypes of LUAD potentially associated with EGFR TKI sensitivity (2), to our knowledge, this is the first predictive model based on quantification of pathology images. Furthermore, to understand the biological mechanisms of EGFR TKI resistance, whether intrinsic (31) or acquired, (32) multiple studies have compared the genetic and proteomic difference among patients, cell lines, or xenografts with different sensitivities to EGFR TKIs. Providing an additional tool, our deep learning–aided quantification strategy enables unbiased analysis to associate phenotypic tumor morphology with underlying biological mechanisms. Since pathological evaluation is the standard of care for most LUAD, our model is universally applicable and thus has the potential to inform treatment decisions as well as drug development.

Although pathology evaluation is part of routine clinical care, digital image analysis is not currently widespread in this setting. In order to translate the proposed image signals into clinical practice, in addition to our effort in facilitating pathology-image analysis through a user-friendly web portal (https://lce.biohpc.swmed.edu/maskrcnn/), we also correlated the image-derived signatures with well acknowledged LUAD subtypes. The finding of correlation between predicted-not-to-benefit group and high-grade subtypes is consistent with a recent observation of prevalence of high-grade subtypes in patients with cancers resistant to EGFR TKI (14).

Crosstalk between tumor cells and fibroblasts has been investigated in vitro (22, 33) and in vivo (24) as a potential therapeutic target and source of EGFR TKI resistance. Our study supports these studies and clinical observations; crosstalk between tumor and fibroblasts cell played role in EGFR TKI resistance. The validity of using increased tumor-stroma interactions as assessed on H&E slides to represent crosstalk between tumor cells and fibroblasts was supported both genetically and phenotypically. Our findings suggest that under the inhibitory effect of an EGFR TKI, HGF-mediated PIP3 activation may bypass EGFR-mediated PIP3 activation, an observation further supported by elevated HGF expression in tumors with higher tumor-stroma interactions. HGF secretion is predominantly attributed to stromal cells (22), but tumor-derived HGF has also been reported in EGFR TKI-resistant tumor cells (28). While it is unclear whether HGF activates MET in a paracrine or autocrine way, elevated tumor-stroma interactions were associated with unfavorable responses to EGFR TKI in clinical practice. Thus, for patients with both sensitizing *EGFR* mutations and extensive tumor-stroma interactions, additionally targeting HGF/MET may restore the response to EGFR TKIs.

In addition, it is interesting to notice that the PD-1 signaling pathway is activated in EGFR TKI–resistant tumors with increased tumor-stroma interaction. This implies that this subgroup of *EGFR*-mutant, TKI resistant patients with LUAD will likely respond to immunotherapy. Since immunotherapy alone is ineffective for *EGFR*-mutant patients with LUAD (34), it will be interesting to further study the potential combination and relationship between TKIs and immunotherapies for patients with activated PD-1 signaling pathway.

### Limitations.

There are some limitations of this study. First, our training and validation LCMC data sets only enrolled patients with *EGFR*-mutant metastatic LUAD, and EGFR TKI therapy was limited to patients with sensitizing *EGFR* mutations. The sample size is relatively small, and mRNA expression data were not available for the LCMC1 or LCMC2 data set, which prevented direct comparison between EGFR TKI survival benefit and transcriptional activities. The cohorts for survival benefitting prediction in the LCMC data sets and biological pathway analysis in the TCGA data set also have different characteristics such as tumor stage distributions. Thus, validation of the proposed model in a larger clinical trial with comprehensive pathological imaging and molecular profiling data would be important. It would confirm the proposed biological mechanism underlying EGFR-TKI resistance and whether PI3K pathway activation predicts a lack of survival benefit from EGFR TKI therapy. Second, due to the nature of retrospective study, some information is not available for more comprehensive evaluations. For example, when and how long EGFR TKIs were administered, and the time of biopsy were not available; therefore, we cannot study how the biopsy time affects the results. Third, this research focuses on inter- rather than intra-tumor heterogeneity and the image features were calculated at the level of the slide and averaged into patient level when 2 or more slides were available for 1 patient. Although the predicted-to-benefit group on the level of the slide mostly agreed with the patient level (Supplemental Figure 8), it would be of interest to study the intra-tumor heterogeneity and how it affects the clinical outcome in future studies.

### Conclusions.

Our prediction model leverages existing routine clinical pathology images to identify patients with *EGFR*-mutant LUAD who are most and least likely to benefit from EGFR TKI therapy. Further, patients likely to be nonbenefitting from EGFR TKI therapy had increased tumor-stroma interactions and showed evidence that the crosstalk between tumor cells and fibroblasts was a source of EGFR TKI resistance. Validation in patients without sensitizing *EGFR* mutations and in larger data sets could inform clinical trials and translate into better outcomes for the overall patient population with LUAD.

## Methods

### Cohorts.

A training cohort was derived from patients who enrolled in the multi-institutional LCMC1 between 2009 and 2012 (12). The training cohort included 168 patients with metastatic LUAD with sensitizing *EGFR* mutations treated with EGFR TKI therapy with available clinical and pathology imaging data. Patients whose pathology images only contained blood (*n* = 12) or stroma (*n* = 6) tissue without tumor cells according to the HD-staining analysis (11) were excluded from the analysis. In total, 177 H&E-stained preEGFR TKI treatment pathology slides from 150 patients — of whom 22 had 2 or more slides available — with *EGFR*-mutant LUAD were used as the training data set to develop a penalized Cox model distinguishing benefitting and nonbenefitting from EGFR TKI treatment. These patients received EGFR TKI treatment, 95.3% were treated with erlotinib, and the corresponding clinical information was available. 

A validation cohort was derived from patients enrolled in the multi-institutional LCMC2 between January 1, 2013 and December 1, 2015 (35). Among all patients enrolled in the LCMC2 study, 127 metastatic patients with LUAD had an *EGFR* mutation detected and had both clinical information and pathology image data available. As with the training cohort, patients whose pathology images only contained blood (*n* = 3) or stroma (*n* = 2) tissue were excluded from the analysis. In total, 131 H&E-stained pathology slides and the corresponding clinical information for 122 patients — of whom 7 had 2 or more slides available — with *EGFR*-mutant LUAD were used as the independent validation data set. Of these, 88 patients carried sensitizing *EGFR* mutations and were treated with EGFR TKI, 88.6% were treated with erlotinib, while the remaining 34 patients did not receive an EGFR TKI treatment. Additionally, 16 patients carried sensitizing *EGFR* mutations and 18 carried other *EGFR* mutations. Although patients with sensitizing *EGFR* mutations typically receive targeted therapy, a variety of factors, including rapid clinical decline after enrollment and loss of follow-up, may have prevented therapeutic intervention. However, the reduced survival of untreated patients was not clearly attributable to early death after enrollment (35). Clinical characteristics of the LCMC1 and LCMC2 data sets were similar, though the LCMC2 cohort had a lower proportion of women and enrolled patients who tended to have more advanced-stage disease relative to the LCMC1 cohort; however, such differences were marginal or not statistically significant (Supplemental Table 4). In both LCMC1 and LCMC2 cohorts, definitive surgery was only done following initial diagnosis and never after the diagnosis of metastatic disease.

An additional 431 40× H&E-stained pathology images for 372 patients with LUAD were acquired from TCGA LUAD data set (https://wiki.cancerimagingarchive.net/display/Public/TCGA-LUAD). Corresponding mutation data and mRNA expression data were collected from the NIH genomic data commons data portal (https://portal.gdc.cancer.gov/projects/TCGA-LUAD). In the TCGA LUAD cohort, 53 patients with *EGFR* mutations were included in the genomic analysis (Supplemental Table 4). The TCGA LUAD cohort was not used for validation, but rather to investigate the relationship between image features and genomics for biological understanding.

### Characterizing the TME.

We used the published HD-staining (11) model, an instance segmentation, deep neural network that was trained to analyze lung cancer pathology images to identify 6 different cell types: tumor cells, stromal cells, lymphocytes, red blood cells, macrophages, and karyorrhexis from H&E-stained images. The HD-staining model was applied to whole pathology slides under 40× magnification (Supplemental Figure 9) and identified the cell type and centroid location of each identified cell nuclei for the purpose of characterizing cell-cell interactions. Pathology images from the LCMC1 and LCMC2 data sets were a mixture of slides captured at 20× and 40×; the images captured at 20× were resized to 40× using a fine–tuned super-resolution generative adversarial network (SR-GAN) (36), after which the HD-staining model was applied. Image regions with tumor nuclei density of at least 10 per 500 × 500 pixel image were classified as tumors (Supplemental Figure 10). Up to one hundred 1,024 × 1,024 pixel-image patch (on average, 83.7 ± 2.6 and 82.6 ± 3.0 patches per slide for the LCMC1 and LCMC2 data sets, respectively), with spatial resolution of 0.25 μm per pixel, were randomly selected from tumor regions of each slide. The cell density, defined as the number of tumor cells detected per one hundred 1,024 × 1,024 pixel-image patch, of each cell type and interactions between tumor cells and their neighbors were extracted for each image patch. Since 6 cell categories were identified by HD-staining in this study, the density of each type of cell was calculated (yielding 6 image features). To quantify the interactions between tumor cells and their neighbors, the cell organization in each image patch was characterized using a Delaunay triangle graph, as previously described (30), which yielded connections between spatially neighboring cells. Cellular interaction between tumor and a specific cell type was defined as the fraction of the cell type neighboring tumor cells among all tumor cell neighbors and calculated using the equation:


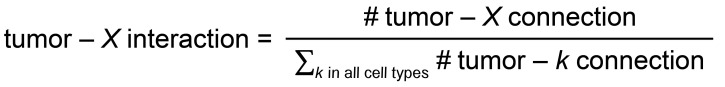


where *X* and *k* refer to 1 of the following cell types: tumor, stroma, lymphocyte, red blood cell, macrophage, or karyorrhexis. For example, the equation quantified tumor-stroma interaction as the ratio of tumor cell–stroma cell connection numbers to the number of connections between tumor cells and all their neighbors; the tumor-stroma interaction was a numeric value that ranged from 0–1 and denoted the percent interaction with stroma cells. The image features were averaged across all image patches extracted from the tumor region; average values were calculated for patients with multiple slides (yielding another 6 image features). In total, 12 image features were extracted for each patient (Supplemental Table 1).

### Development and validation of the EGFR TKI survival benefit prediction model.

OS, defined as the date of diagnosis of metastatic disease till death or last contact, was used to evaluate the benefit from EGFR TKI treatment for survival analyses. A penalized Cox proportional hazards (CoxPH) model (13) for OS was developed using the LCMC1 data set to correlate benefits form EGFR TKI treatment with image features. Since there are correlations among the image features (Supplemental Figure 11), an elastic net penalty was used to avoid overfitting and to select the final 2 most predictive features from the 12 input image features (Supplemental Table 1). The survival benefit prediction model calculated a risk score for 1 patient by summing the products between features and corresponding coefficients; a higher risk score indicated that the individual was predicted-not-to-benefit from TKI treatment. To validate the survival benefit prediction model, risk scores were calculated for the LCMC2 cohort based on the prediction model derived from the LCMC1 cohort. Patients in LCMC2 were divided into 2 groups using a median split, EGFR TKI predicted-to-benefit or predicted-not-to-benefit groups.

### Association analysis between image features and gene expression of biological pathways.

To identify potential biological mechanisms underlying relationships between image features and EGFR TKI treatment benefit, gene expression data of 53 patients with *EGFR* mutant LUAD from the TCGA data set were collected and preprocessed: genes whose mRNA expression levels were 0 in more than 20% of patient samples were removed. Correlations between mRNA expression levels and image-derived cellular interactions were evaluated using Spearman’s rank correlations. GSEA was performed based on the Spearman’s rank correlation coefficients for each image-derived cellular interaction as determined by the prediction model, with gene sets derived from the Reactome, KEGG, and GO databases (37).

### Data availability.

Pathology images that support the findings of this study were available online in TCGA LUAD (https://wiki.cancerimagingarchive.net/display/Public/TCGA-LUAD). mRNA expression data for the TCGA data set were available online from TGCA LUAD (https://portal.gdc.cancer.gov/projects/TCGA-LUAD).

### Statistics.

Survival differences were visualized using the Kaplan-Meier method and estimated using log-rank test. HRs and CIs were estimated using Cox proportional hazards models. The interaction between the predicted groups and EGFR TKI treatment was evaluated using a multivariate Cox proportional hazards model after adjusting for other clinical variables, including age, sex, smoking status, and surgical resection. R software, version 3.4.2, and R packages (survival, version 2.41-3; glmnet, version 2.0-13; spatstat, version 1.55-1) were used for the survival analysis (38, 39). GSEA *P* values were adjusted using the Benjamini-Hochberg procedure. Two-sided *P* values of less than 0.05 were considered significant. R packages Hmisc (version 4.1-1), fgsea (version 1.4.1), and gplots (version 3.0.1) were used for the image-genomic association analysis (40).

### Study approval.

IRB approval was obtained from all institutions enrolling patients in LCMC1, LCMC2, and TCGA (12, 35, 41).

## Author contributions

SW, YX, and GX designed the study. SW and RR implemented and trained the neural network model. SW and LC performed genomic analysis. SW, RR, DMY, JF, JAB, SY, LC, CB, YX, JM, and GX analyzed the data and wrote the manuscript. JF, JAB, and SY provided critical inputs as lung cancer pathologists. IIW, PAB, JM, and MGK provided the LCMC data sets and supervised the study. SW, RR, DMY, JF, JAB, SY, LC, CB, LDB, CW, DA, LS, BEJ, DJK, IIW, PAB, JM, GX, MGK, and YX read and commented on the manuscript.

## Supplementary Material

Supplemental data

## Figures and Tables

**Table 1:**
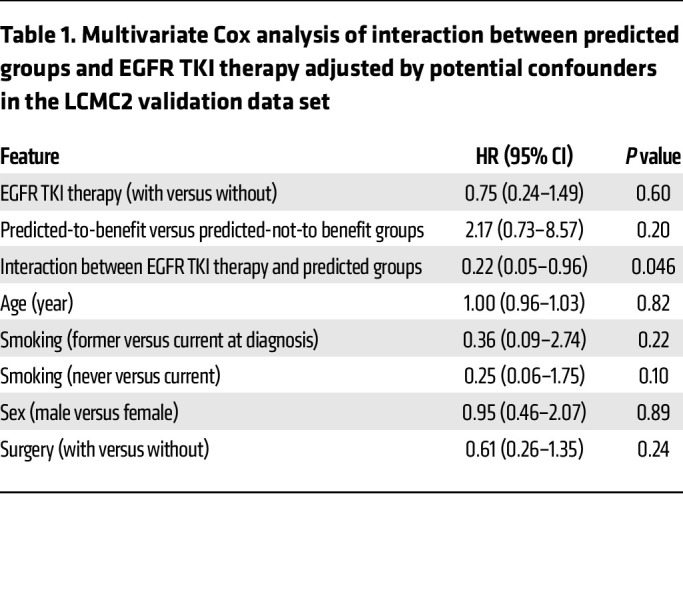
Multivariate Cox analysis of interaction between predicted groups and EGFR TKI therapy adjusted by potential confounders in the LCMC2 validation data set

## Version 1 01/17/2023

Electronic publication

## References

[1] Cohen MH (2005). FDA drug approval summary: Erlotinib (Tarceva (R)) tablets. Oncologist.

[2] Santos GD (2011). EGFR mutations and lung cancer. Annu Rev Pathol-Mech.

[3] Maemondo M (2010). Gefitinib or chemotherapy for non-small-cell lung cancer with mutated EGFR. N Engl J Med.

[4] Zhou C (2011). Erlotinib versus chemotherapy as first-line treatment for patients with advanced EGFR mutation-positive non-small-cell lung cancer (OPTIMAL, CTONG-0802): a multicentre, open-label, randomised, phase 3 study. Lancet Oncol.

[5] Rosell R (2012). Erlotinib versus standard chemotherapy as first-line treatment for European patients with advanced EGFR mutation-positive non-small-cell lung cancer (EURTAC): a multicentre, open-label, randomised phase 3 trial. Lancet Oncol.

[6] Kim YH (2004). Dominant papillary subtype is a significant predictor of the response to gefitinib in adenocarcinoma of the lung. Clin Cancer Res.

[7] Miller VA (2004). Bronchioloalveolar pathologic subtype and smoking history predict sensitivity to gefitinib in advanced non-small-cell lung cancer. J Clin Oncol.

[8] Yatabe Y (2005). EGFR mutation is specific for terminal respiratory unit type adenocarcinoma. Am J Surg Pathol.

[9] LeCun Y (2015). Deep learning. Nature.

[10] Wang S (2019). Artificial intelligence in lung cancer pathology image analysis. Cancers (Basel).

[11] Wang S (2020). Computational staining of pathology images to study the tumor microenvironment in lung cancer. Cancer Res.

[12] Sholl LM (2015). Multi-institutional oncogenic driver mutation analysis in lung adenocarcinoma: the lung cancer mutation consortium experience. J Thorac Oncol.

[13] Simon N (2011). Regularization paths for cox’s proportional hazards model via coordinate descent. J Stat Softw.

[14] Yu X (2021). Adenocarcinoma of high-grade patterns associated with distinct outcome of first-line chemotherapy or egfr-tkis in patients of relapsed lung cancer. Cancer Manag Res.

[15] Wu JY (2016). Association between tumor-stroma ratio and prognosis in solid tumor patients: a systematic review and meta-analysis. Oncotarget.

[16] Hou HL (2019). Concurrent TP53 mutations predict poor outcomes of EGFR-TKI treatments in Chinese patients with advanced NSCLC. Cancer Manag Res.

[17] Henke E (2020). Extracellular matrix in the tumor microenvironment and its impact on cancer therapy. Front Mol Biosci.

[18] Liu LH (2021). Tumor immune microenvironment in epidermal growth factor receptor-mutated non-small cell lung cancer before and after epidermal growth factor receptor tyrosine kinase inhibitor treatment: a narrative review. Transl Lung Cancer R.

[19] Parra HS (2004). Analysis of epidermal growth factor receptor expression as a predictive factor for response to gefitinib (‘Iressa’, ZD1839) in non-small-cell lung cancer. Brit J Cancer.

[20] Ortiz-Zapater E (2017). MET-EGFR dimerization in lung adenocarcinoma is dependent on EGFR mtations and altered by MET kinase inhibition. PLoS One.

[21] Engelman JA (2005). ErbB-3 mediates phosphoinositide 3-kinase activity in gefitinib-sensitive non-small cell lung cancer cell lines. Proc Natl Acad Sci U S A.

[22] Matsumoto K, Nakamura T (2006). Hepatocyte growth factor and the Met system as a mediator of tumor-stromal interactions. Int J Cancer.

[23] Wang W (2009). Crosstalk to stromal fibroblasts induces resistance of lung cancer to epidermal growth factor receptor tyrosine kinase inhibitors. Clin Cancer Res.

[24] Yi YM (2018). Cancer-associated fibroblasts promote epithelial-mesenchymal transition and EGFR-TKI resistance of non-small cell lung cancers via HGF/IGF-1/ANXA2 signaling. Biochim Biophys Acta Mol Basis Dis.

[25] Byers LA (2013). An epithelial-mesenchymal transition gene signature predicts resistance to EGFR and PI3K inhibitors and identifies Axl as a therapeutic target for overcoming EGFR inhibitor resistance. Clin Cancer Res.

[26] Thomson S (2005). Epithelial to mesenchymal transition is a determinant of sensitivity of non-small-cell lung carcinoma cell lines and xenografts to epidermal growth factor receptor inhibition. Cancer Res.

[27] Yauch RL (2005). Epithelial versus mesenchymal phenotype determines in vitro sensitivity and predicts clinical activity of erlotinib in lung cancer patients. Clin Cancer Res.

[28] Uramoto H (2011). Expression of selected gene for acquired drug resistance to EGFR-TKI in lung adenocarcinoma. Lung Cancer.

[29] Larsen JE (2016). ZEB1 drives epithelial-to-mesenchymal transition in lung cancer. J Clin Invest.

[30] Wang S (2019). Computational staining of pathology images to study the tumor microenvironment in lung cancer. Cancer Res.

[31] Santoni-Rugiu E (2019). Intrinsic resistance to EGFR-tyrosine kinase inhibitors in *EGFR*-mutant non-small cell lung cancer: differences and similarities with acquired resistance. Cancers (Basel).

[32] Westover D (2018). Mechanisms of acquired resistance to first- and second-generation EGFR tyrosine kinase inhibitors. Ann Oncol.

[33] Choe C (2015). Crosstalk with cancer-associated fibroblasts induces resistance of non-small cell lung cancer cells to epidermal growth factor receptor tyrosine kinase inhibition. Onco Targets Ther.

[34] Yu S (2018). Immunotherapy strategy of EGFR mutant lung cancer. Am J Cancer Res.

[35] Aisner DL (2018). The impact of smoking and TP53 mutations in lung adenocarcinoma patients with targetable mutations-the lung cancer mutation consortium (LCMC2). Clin Cancer Res.

[37] Fabregat A (2016). The Reactome pathway Knowledgebase. Nucleic Acids Res.

[38] https://CRAN.R-project.org/package=survival.

[39] https://www.R-project.org/.

[41] Kris MG (2014). Using multiplexed assays of oncogenic drivers in lung cancers to select targeted drugs. JAMA.

