# Supplementary material for: Features of tumor-microenvironment images predict targeted therapy survival benefit in patients with EGFR-mutant lung cancer
Source: J Clin Invest. 2023 Jan 17;133(2):e160330. doi: 10.1172/JCI160330 (PMC9843059; doi:10.1172/JCI160330)

Supplemental Tables and Figures

Supplemental Table 1 Variable coefficients in the epidermal growth factor receptor (EGFR) tyrosine kinase inhibitor (TKI) treatment survival benefit prediction model. Since an elastic net based penalty was used in this model for variable selection, majority of features has coefficients of 0 and only two variables were selected.

Features	Coefficients
Tumor nuclei density	.
Stroma nuclei density	.
Lymphocyte density	.
Red blood cell density	.
Macrophage density	.
Karyorrhexis density	.
Tumor-tumor interaction	-1.082
Tumor-stroma interaction	0.407
Tumor-lymphocyte interaction	.
Tumor-red blood cell interaction	.
Tumor-macrophage interaction	.
Tumor-karyorrhexis interaction	.

Supplemental Table 2 Association between predicted benefitting groups and lung adenocarcinoma subtypes annotated by pathologists.

Training (LCMC1)	Acinar	Papillary	Micro-papillary	Solid
Benefitting	55 (75.3%)	6 (8.2%)	1 (1.4%)	11 (15.1%)
Non-benefitting	28 (50.0%)	1 (1.8%)	0 (0.0%)	27 (48.2%)

Validation (LCMC2)	Acinar	Lepidic	Solid
Benefitting	30 (61.2%)	2 (4.1%)	17 (34.7%)
Non-benefitting	15 (28.3%)	0 (0.0%)	38 (71.7%)

Supplemental Table 3. Visualization of the pathology patterns associated with predictive features. Four patients from the benefitting group and non-benefitting group were selected, and their predictive image features, survival outcomes, and pathological region of interest were listed. TSR, tumor-stroma ratio.

Predictive features			TSR	Survival outcomes		Pathology Image
t-t interaction	t-s interaction	Risk score		OS (years)	Death	
Predicted benefitting group						
0.69	0.15	-0.68	2.85	3.4	1	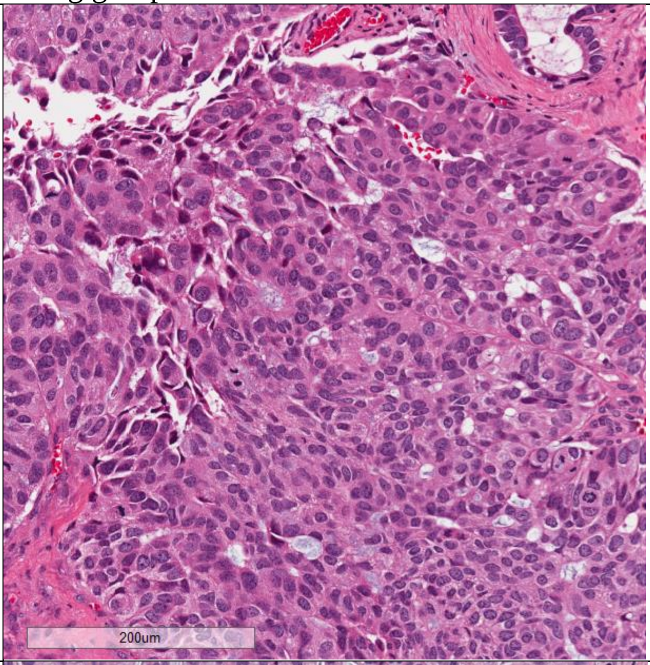
0.67	0.13	-0.67	3.78	2.7	1	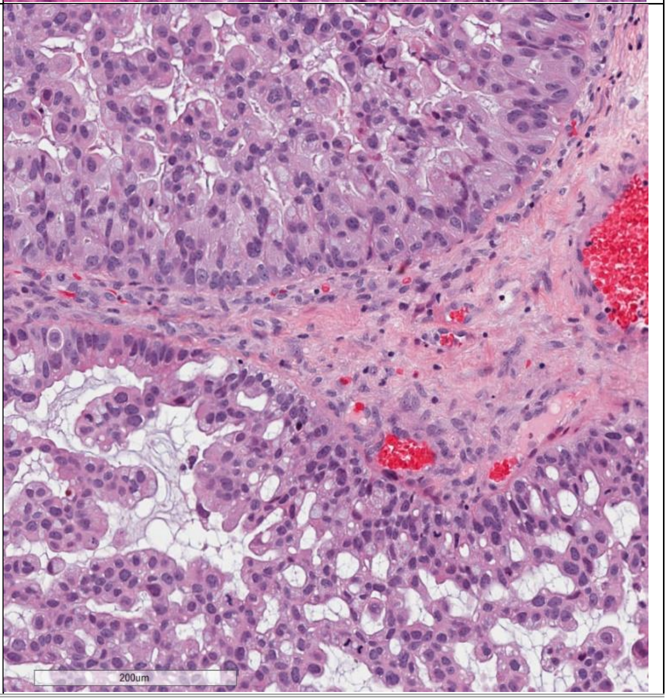

Predicted non-benefitting group						
0.21	0.36	-0.08	0.27	0.81	1	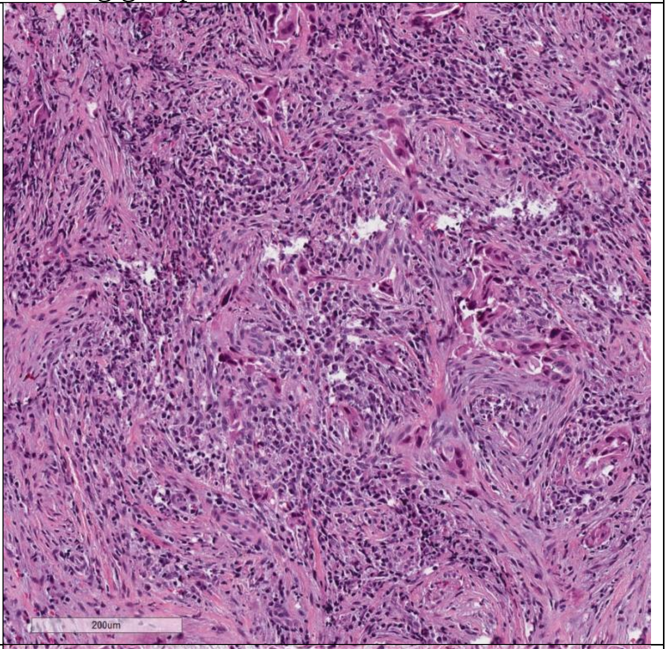
0.20	0.33	-0.08	0.35	0.61	1	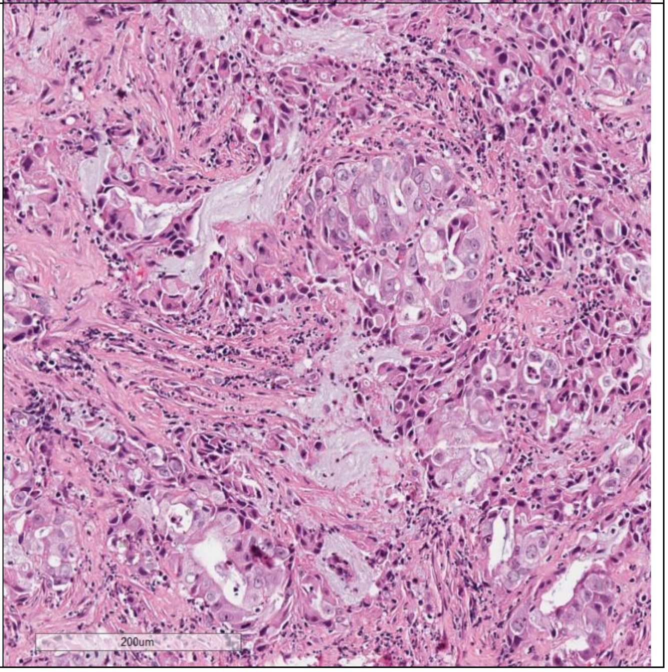

Supplemental Table 4 Patient characteristics of the training, validation, and TCGA lung adenocarcinoma datasets.

LCMC, Lung Cancer Mutation Consortium; LUAD, lung adenocarcinoma; OS, overall survival; TCGA, the Cancer Genome Atlas; TKI, tyrosine kinase inhibitor. P values are calculated using Chi-Square Tests for categorical variables and student's t-test for continuous variables.

	**Training (LCMC1)**	**Validation (LCMC2)**	***P* value**	**Genomic analysis (TCGA LUAD)**
**# *EGFR* mutated (n)**	150	122		53
**# with available OS**	141	118		-
**# EGFR TKI treated (n, %)**	150 (100%)	88 (72%)		2 yes, 20 no, 31 unknown
**# with available OS in treated group**	141	87		-
**# Biopsy slides**	178	132		68
**Age (year** ± standard deviation**)**	61.5 ± 10.5	62.4 ± 10.3	0.53	65.2 ± 8.9
**Gender (n, %)**			0.06	
**Male**	33 (22%)	40 (33%)		17 (32%)
**Female**	117 (78%)	82 (67%)		36 (68%)
**Smoking status (n, %)**			0.19	
**Current**	1 (1%)	3 (2%)		9 (17%)
**Former**	56 (37%)	54 (44%)		22 (42%)
**Never**	93 (62%)	64 (52%)		22 (42%)
**Unknown**	0 (0%)	1 (1%)		0 (0%)
**Surgery received (n, %)**			0.23	
**No**	87 (58%)	79 (65%)		0 (0%)
**Yes**	63 (42%)	41 (34%)		53 (100%)
**Unknown**	0 (0%)	2 (1%)		0 (0%)
**Stage at initial diagnosis (n, %)**			0.05	
**I**	13 (9%)	5 (4%)		27 (51%)
**II**	8 (5%)	3 (2%)		15 (28%)
**III**	23 (15%)	10 (8%)		6 (11%)
**IV**	104 (69%)	101 (83%)		5 (9%)
**Unknown**	2 (1%)	3 (2%)		0 (0%)

Supplemental Figure 1. Visualization of epidermal growth factor receptor (EGFR) tyrosine kinase inhibitor (TKI) survival benefit prediction model. Distribution of risk scores from the Lung Cancer Mutation Consortium 2 (LCMC2) dataset was shown in white circle. Lighter color indicates less likely to benefit from EGFR TKI therapy.

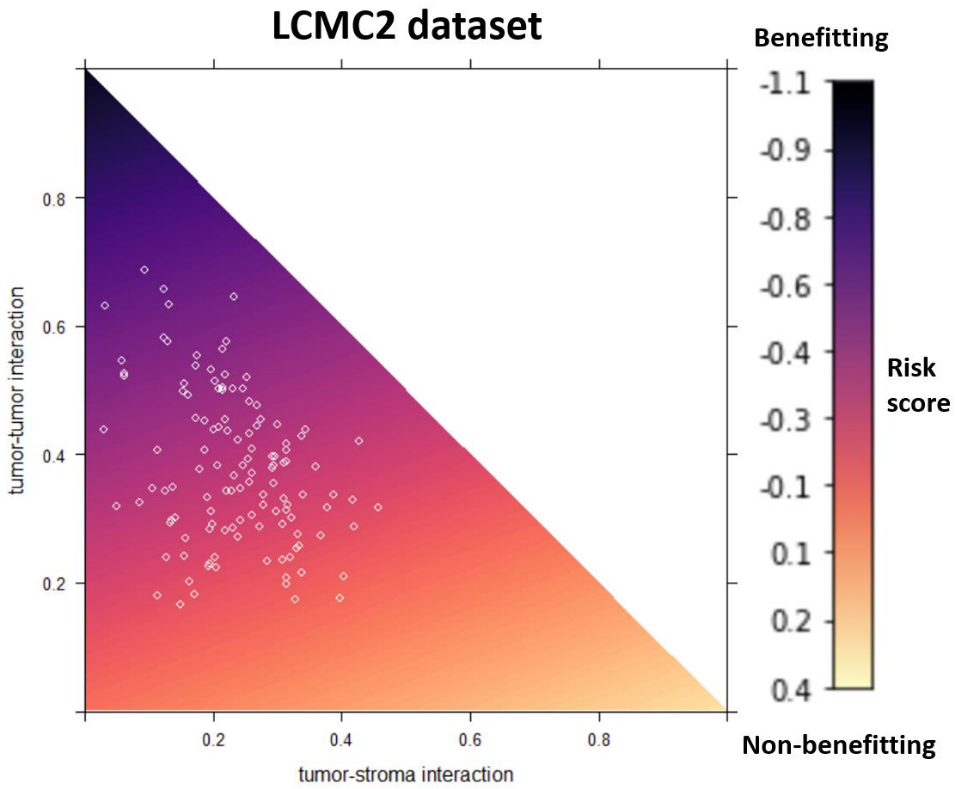

Supplemental Figure 2. Comparison among patient groups stratified by predicted benefitting group, epidermal growth factor receptor (EGFR) tyrosine kinase inhibitor (TKI) treated (Ttx, w+) or not (wo), and *EGFR* mutation type. sEGFR, sensitizing *EGFR* mutation; oEGFR, other *EGFR* mutation; Ttx, targeted therapy.

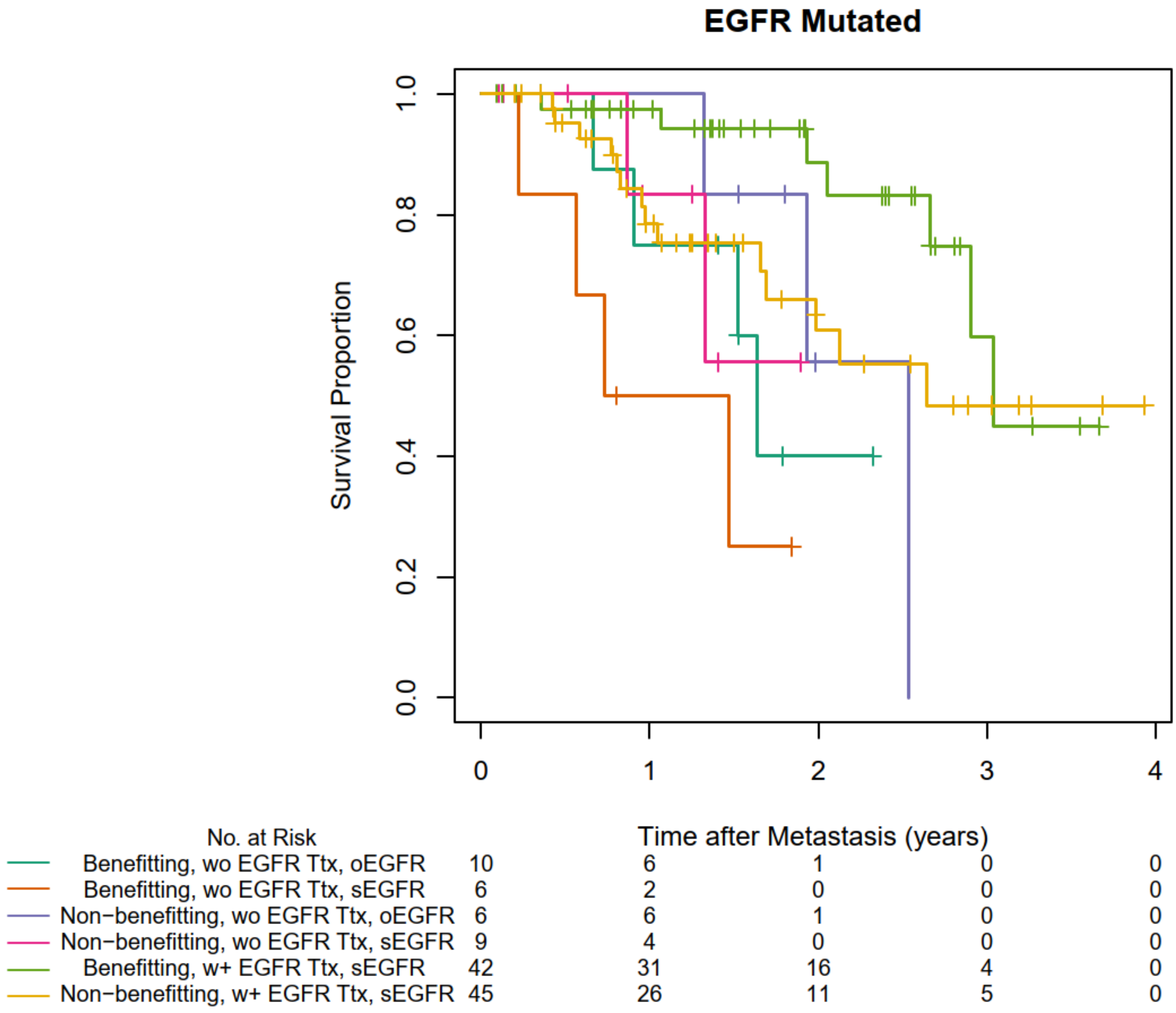

Supplemental Figure 3 Association between tumor-stroma ratio (TSR) and predicted risk score, benefitting group, and overall survival. (A) Scatterplot between TSR and predicted risk score. **(B)** Boxplot between TSR and predicted benefitting group**. (C)** Multivariate survival analysis of TSR groups adjusted by potential confounders.

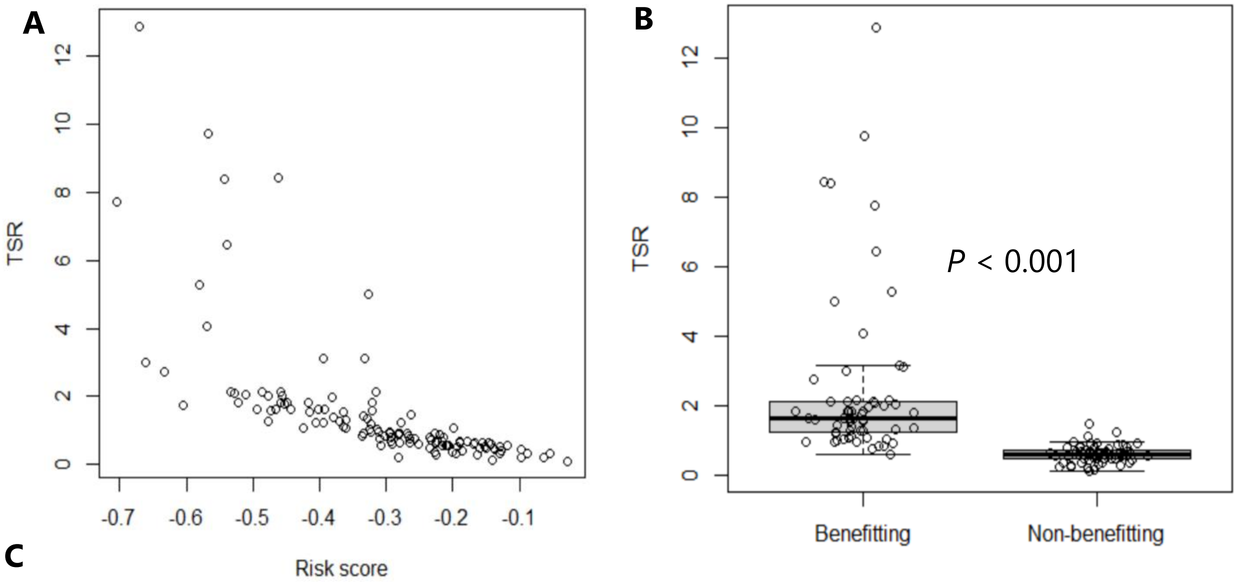

C

Feature	HR (95% CI)	p value
EGFR TKI therapy (with vs. without)	0.19 (0.06 – 0.61)	0.0051
TSR (low vs. high)	0.52 (0.15 – 1.82)	0.31
Interaction between EGFR TKI therapy and TSR	3.32 (0.74 – 14.97)	0.12
Age (per year)	1.00 (0.96 – 1.03)	0.82
Smoking (former vs. current)	0.52 (0.09 – 3.04)	0.47
Smoking (never vs. current)	0.33 (0.06 – 1.87)	0.21
Gender (male vs. female)	1.04 (0.49 – 2.19)	0.92
Surgery (with vs. without)	0.67 (0.31 – 1.44)	0.31

Supplemental Figure 4. KEGG and GO analysis results of gene sets of interest that best match the Reactome genes sets in Figure 5. (A) GSEA analysis results in the KEGG pathways. **(B)** GSEA analysis results for the GO gene sets.

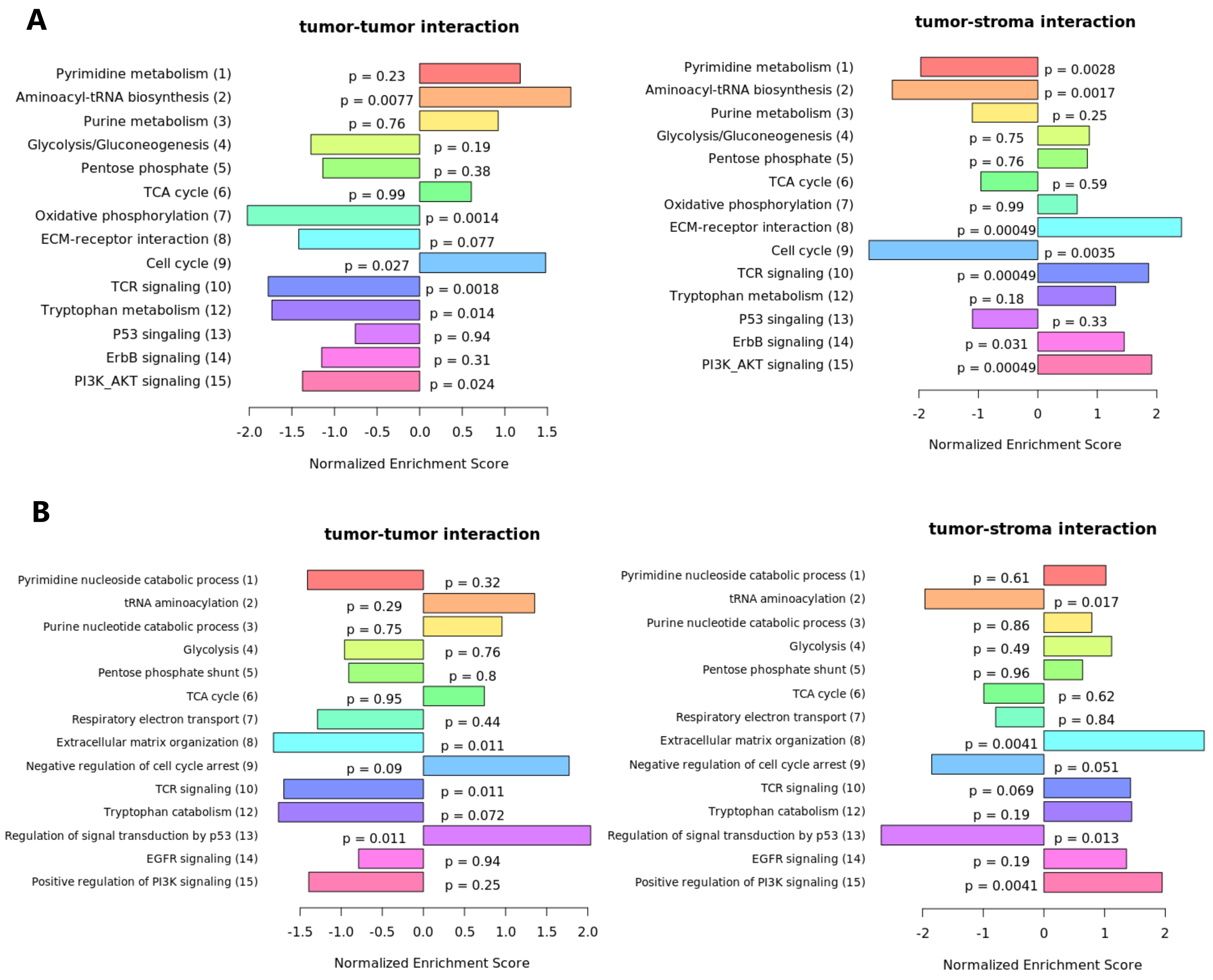

Supplemental Figure 5. Negative control gene set enrichment analysis (GSEA) with randomized patients correlating mRNA expression with tumor-tumor interaction (left) and tumor-stroma interaction (right)

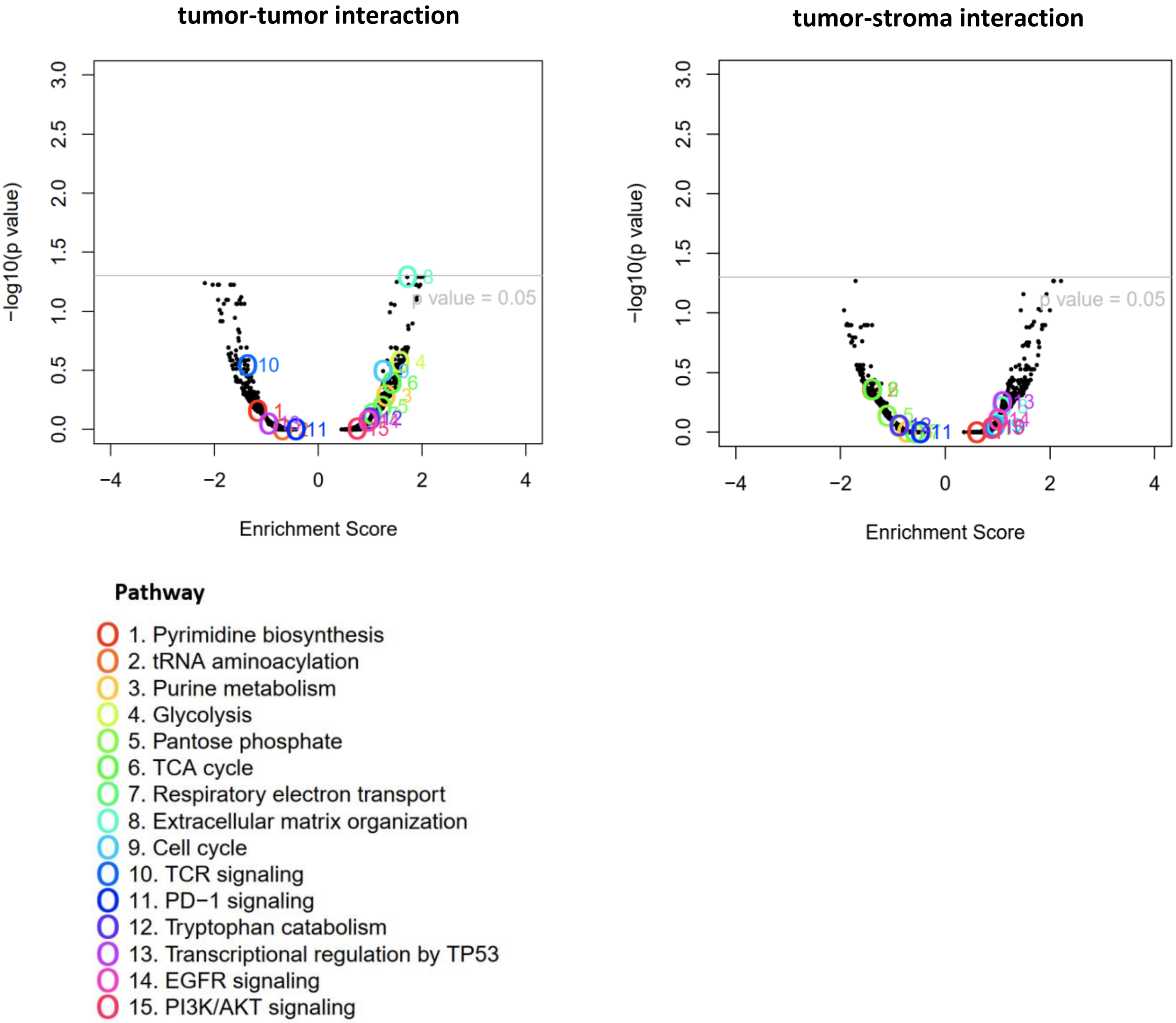

Supplemental Figure 6. Illustration of MET- and EGFR-mediated PIP3 activation pathways. Figures are recreated from the Reactome database, available at https://reactome.org/PathwayBrowser/#/R-HSA-6806834 (left) and https://reactome.org/PathwayBrowser/#/R-HSA-177929&SEL=R-HSA-177929 (right). HGF, hepatocyte growth factor; EGF, epidermal growth factor; EGFR, epidermal growth factor receptor.

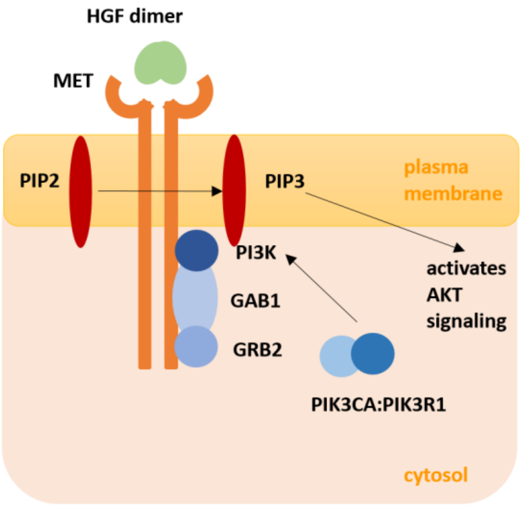

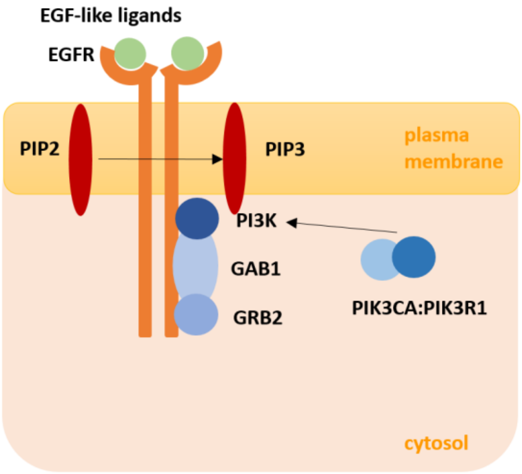

Supplemental Figure 7. DNA methylation heatmap depicting relationships between tumor-stroma interactions and methylation status on the transcriptional start site (TSS). Patients with *EGFR* mutation in the TCGA dataset were grouped and sorted according to tumor-stroma interaction, as each column depicts one patient group.

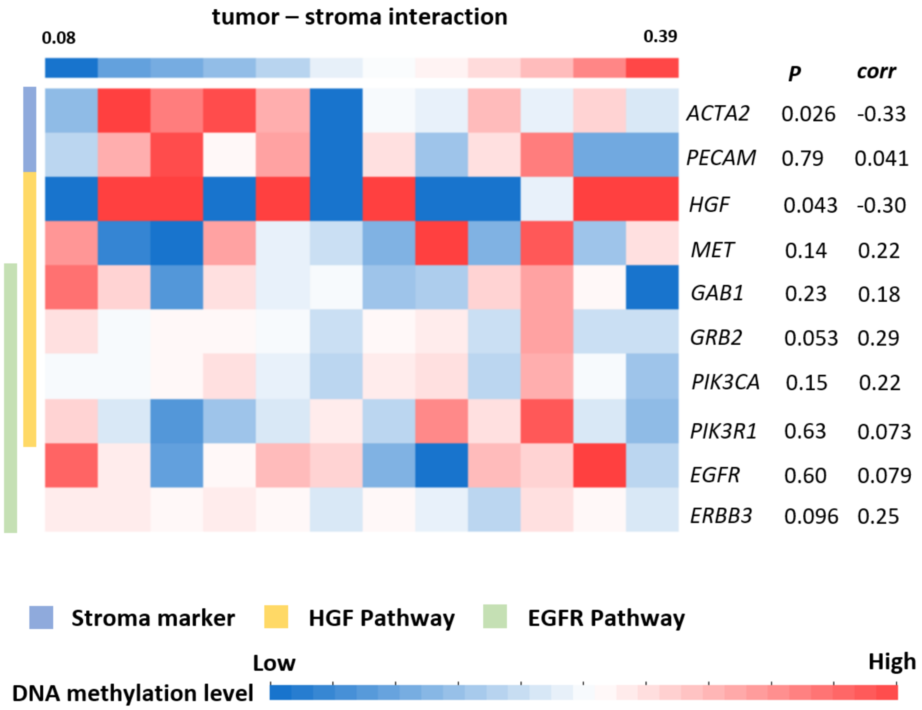

Supplemental Figure 8. Concordance between patient- and slide-level predictions when two for more slides are available for a single patient. Blue, predicted as benefitting from EGFR TKI treatment; orange, predicted as non-benefitting from EGFR TKI treatment.

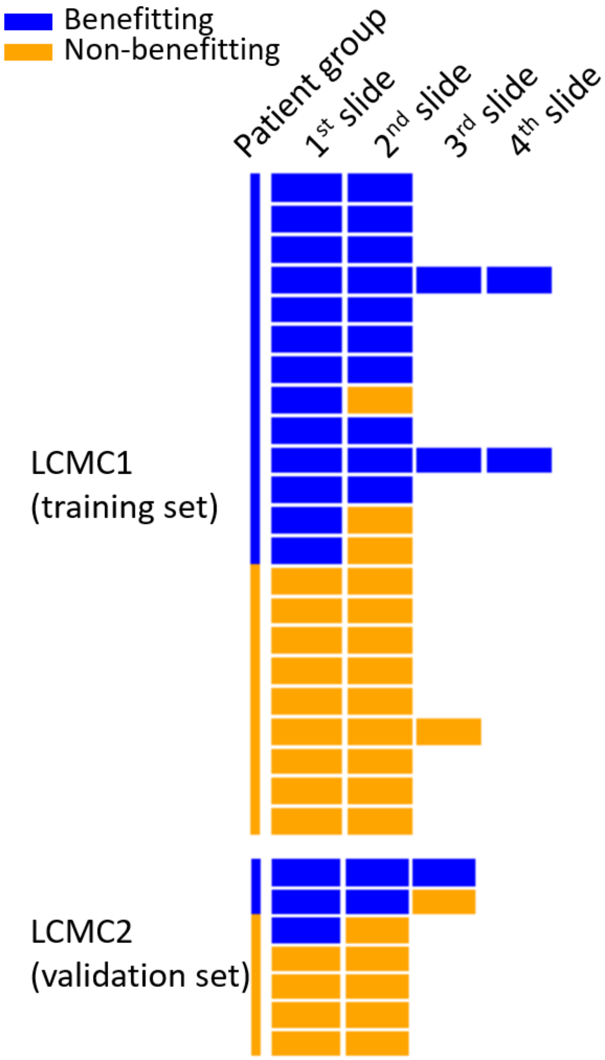

Supplemental Figure 9. Example of whole-slide HD-Staining.

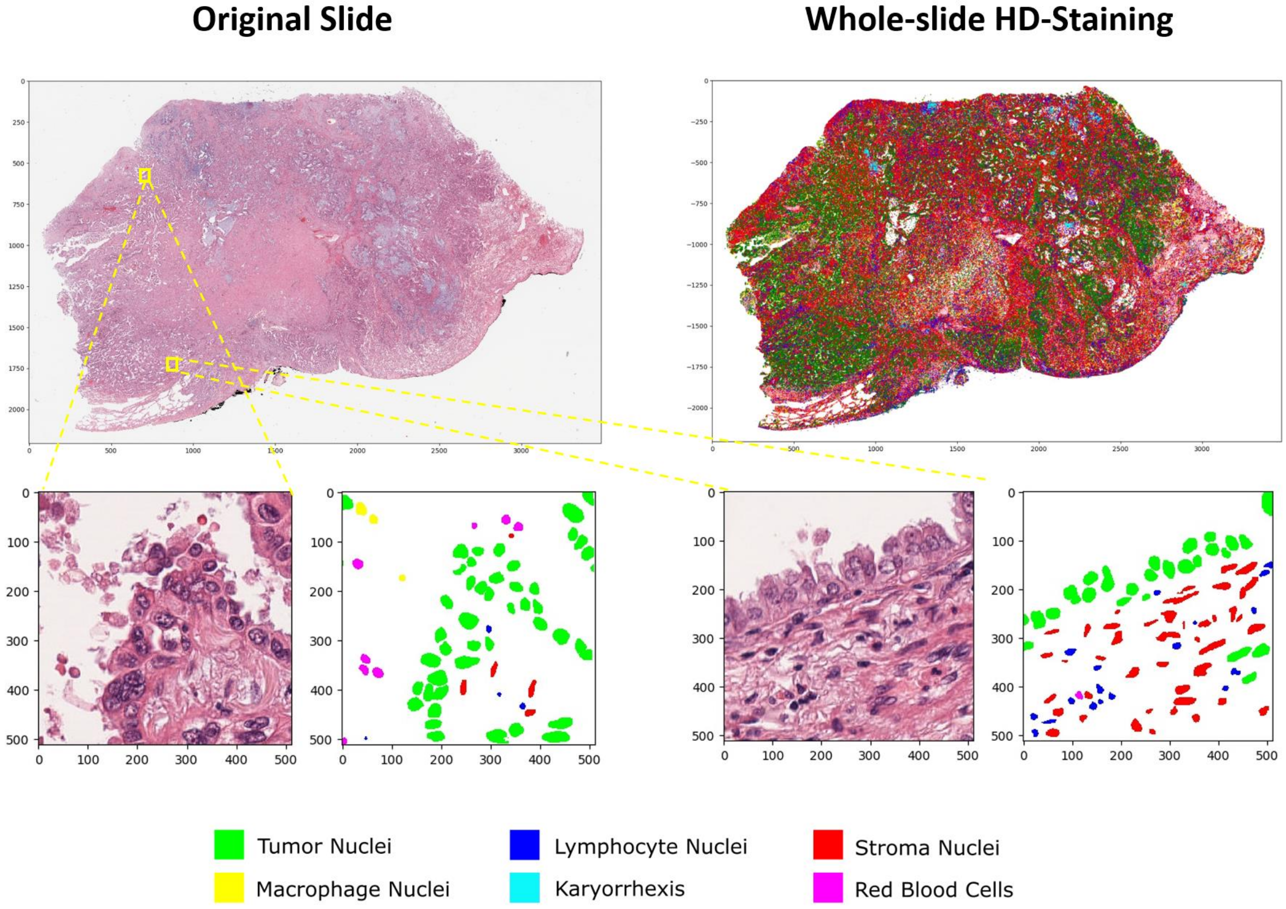

Supplemental Figure 10. Flowchart of automatic tumor region detection. Each pixel in the tumor cell density plot corresponds to a 500 × 500 pixel image patch under 40X magnification. Regions with tumor nuclei (stained in green in the plot of HD-Staining output) density ≥ 10 per 500 × 500 pixel image patches were classified as tumors (white region in the tumor region detection plot).

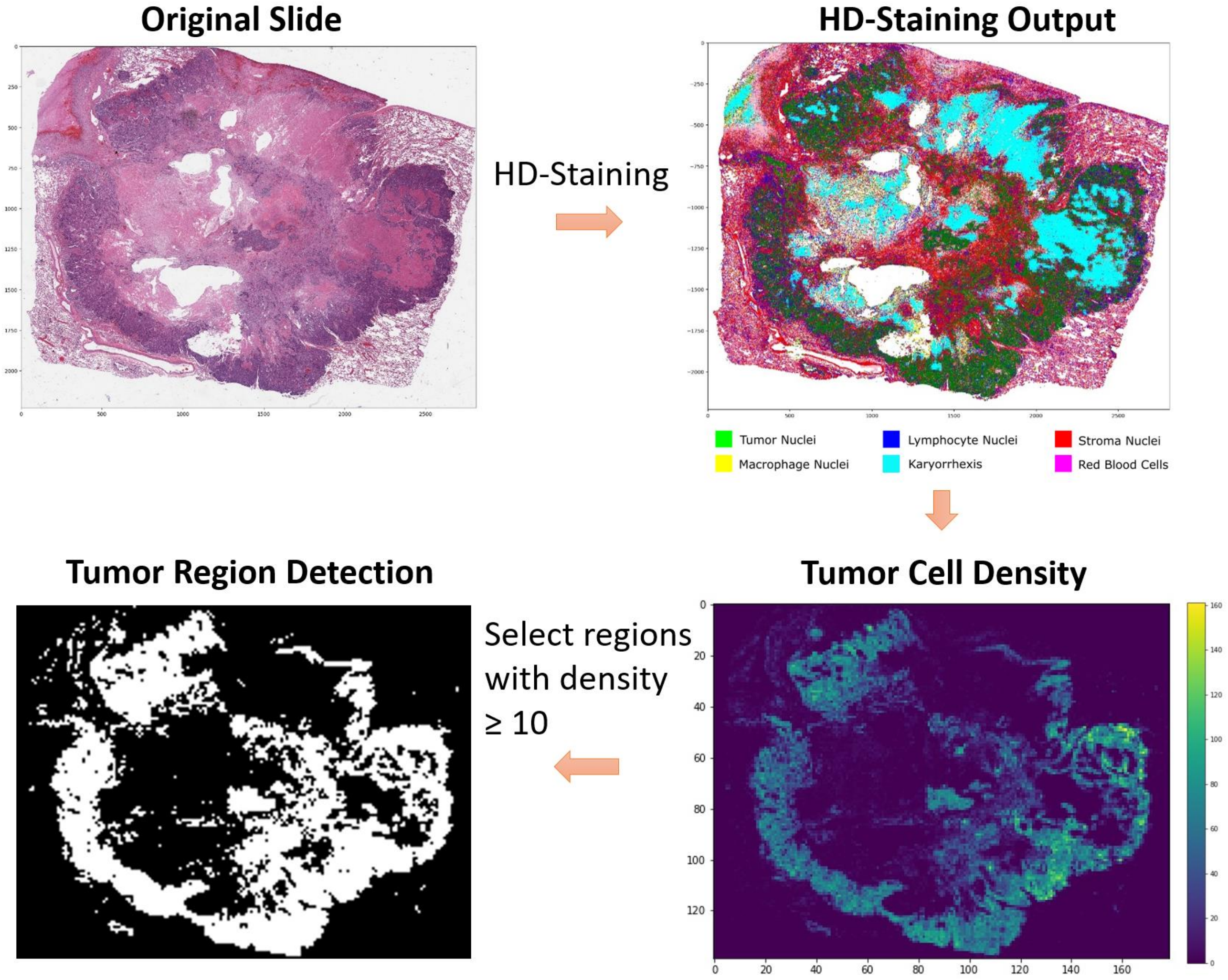

Supplemental Figure 11. Scatterplots of correlations among tumor-tumor interaction, tumor-stroma interaction, tumor cell density, and stroma cell density in the LCMC1 training set.

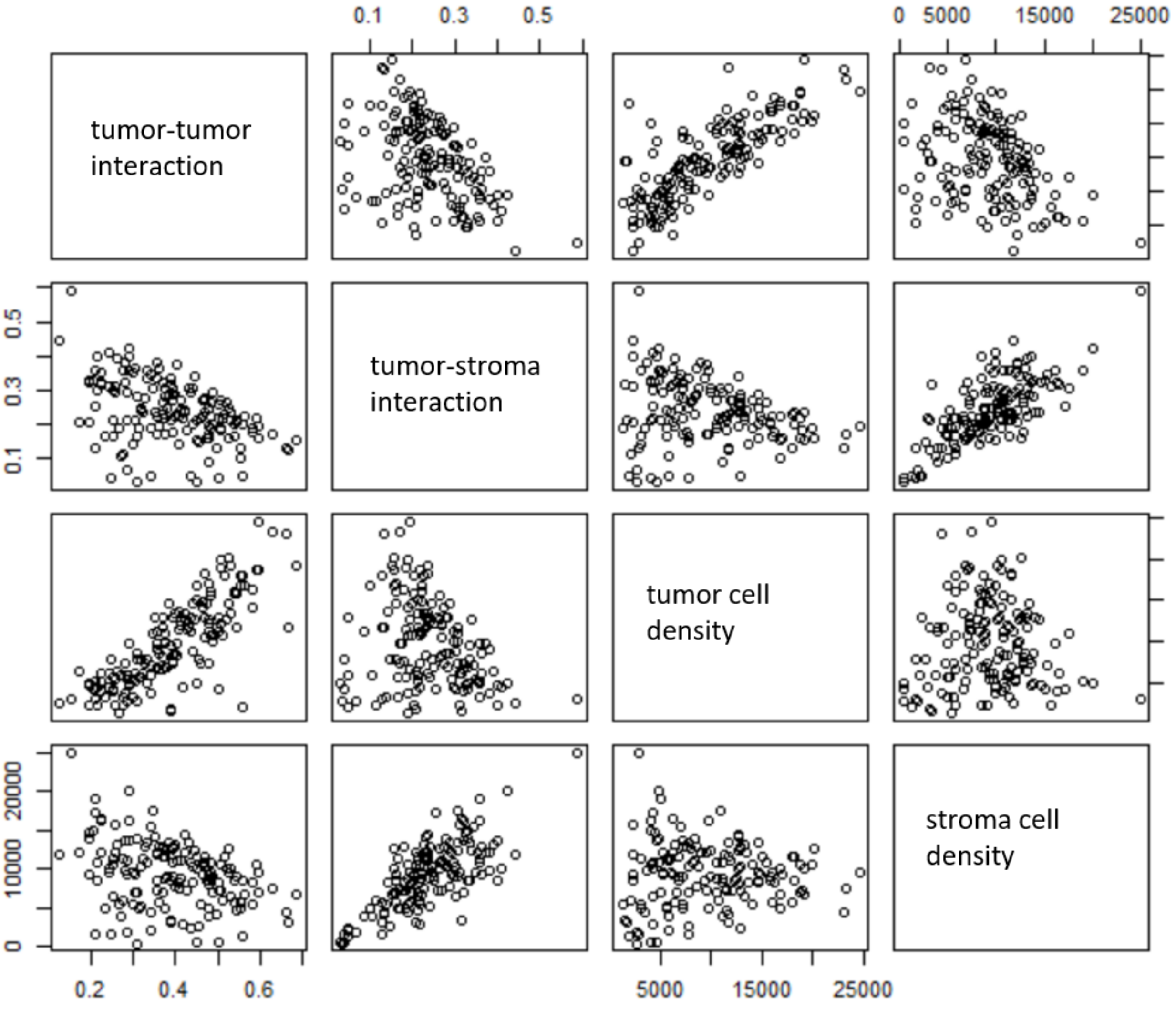

Supplement: Supplemental data [file jci-133-160330-s133.pdf]
